# Prevalence of Coronary Artery Disease and the Associated Risk Factors in the Adult Population of Borujerd City, Iran

**Published:** 2019-01

**Authors:** Ali Maleki, Reza Ghanavati, Mahdi Montazeri, Saeid Forughi, Behjat Nabatchi

**Affiliations:** 1 *Madani Heart Center, Lorestan University of Medical Sciences, Khorramabad, Iran.*; 2 *Imam Khomeini Hospital, Lorestan University of Medical Sciences, Khorramabad, Iran.*; 3 *School of Nursing, Lorestan University of Medical Sciences, Khorramabad, Iran.*

**Keywords:** *Prevalence*, *Coronary artery disease*, *Risk factors*, *Iran*

## Abstract

**Background:** Cardiovascular events are the leading cause of mortality and are highly associated with lifestyle. We aimed to evaluate the prevalence of coronary artery disease (CAD) and its major risk factors in the western Iranian city of Borujerd.

**Methods:** This cross-sectional study was conducted on 801 subjects older than 35 years of age, recruited via cluster sampling in Borujerd. The diagnosis of CAD was based on the positive results of Rose Angina Questionnaire, Minnesota coding, or prior history of CAD. Then, the risk factors were measured by biochemistry and relevant laboratory examinations, or data extraction from the subjects’ history.

**Results: **The study sample consisted of 412 men and 389 women at a mean age of 54.82±12.11 years. The prevalence of risk factors including hypertension, diabetes mellitus, dyslipidemia, smoking, and obesity was 38.2%, 17.4%, 64%, 23.2%, and 22.8%, respectively. Based on the criteria, 19.1% and 31.7% of the CAD cases were definite and probable, respectively. Furthermore, 12.5% had definite signs and symptoms of CAD, and 5.4% had positive Rose Angina Questionnaire outcomes.

**Conclusion:** The current study demonstrated the distribution of CAD in the Iranian city of Borujerd and it was demonstrated that obesity and smoking are the most common risk factors, respectively.

## Introduction

Cardiovascular diseases (CVDs) are a class of diseases that involve the heart or blood vessels and leading cause of mortality in developed countries.^1^ Major atherosclerotic diseases including coronary artery disease (CAD) are multifactorial and highly associated with lifestyle.^1^^, ^^2^

At the beginning of the 20th century, the mortality rate of CVDs was estimated at 10%, while in the 2000s, it has already risen to 50% and 25% in developed and developing countries, respectively. As researchers have estimated, 25 million people will suffer from CVDs in 2020, and this group of diseases will be the main cause of death worldwide (about 30%).^1^^-^^3^ The prevalence of CVDs varies from one country to another; all health-care providers should, therefore, evaluate the associated risk factors and mortality and morbidity rates to improve the general health of the population.

Non-preventable (e.g., age, sex, and race) and preventable (e.g., lifestyle and blood pressure) risk factors for CVDs have been introduced in previous studies. Researchers have demonstrated that 8 out of 9 preventable risk factors are somehow related to dietary patterns. Consequently, to reduce CVD risk factors, investigators have made various efforts such as adding omega-3 fatty acids to diets (even low-fat diets).^4^

With an explosion-like increase in urbanization and unhealthy lifestyles in developing countries, the rate of CVDs has significantly increased. Considering the significant economic burden and variations in risk factors in different societies, several population-based studies have been conducted to determine and evaluate CVD risk factors.^5^^-^^7^

In the Iranian province of Lorestan, a campaign called "the Healthy Heart Program" has been launched. Risk factor assessment is one of the most important parts of the program. Therefore, the aim of the present study was to evaluate the prevalence of CVDs and their major risk factors in the western Iranian city of Borujerd, one of the most prominent cities in Lorestan Province.

## Methods

The patients were fully informed about the study, and written consent forms were obtained prior to evaluation. In total, 795 volunteers were selected from the Borujerd population. For sampling purposes, different regions of Borujerd were divided into 27 clusters, comprising 16 urban and 9 rural clusters, according to the municipal district division. Given the actual number of 35-year-olds (and above) in each cluster, 30-31 residents of Borujerd were selected. 

The diagnosis of CAD was based on the positive results of one of the following scales: Rose Angina Questionnaire, Minnesota coding, or prior history of CAD (i.e., history of coronary care unit (CCU) admission or any medical file indicating CAD such as angiography or exercise-stress-test results). To this end, 2 cardiologists, 4 general practitioners, and 6 health technicians were hired to evaluate all the participants, using 2 questionnaires. All demographic data including age, sex, and anthropometric measurements were collected. Subsequently, the standard Rose Angina Questionnaire was used for the assessment of angina pectoris, and the Minnesota coding was applied for electrocardiographic (ECG) analysis. 

 The subjects were classified into 3 groups: (1) non-CAD, (2) probable CAD, and (3) definite CAD (definite Minnesota ECG codes, typical chest pain in the Rose Angina Questionnaire, or medical records indicating a previous diagnosis of CAD). Then the risk factors were measured by biochemistry or the relevant laboratory examinations or extracted from the subjects’ history.

Complete cardiovascular examinations, consisting of blood-pressure evaluation, auscultation, and jugular venous pressure assessment, along with conventional 12-lead ECG study (Minnesota coding) and biochemical profiling including the assessment of serum glucose, cholesterol, triglyceride, high-sensitivity C-reactive protein, low-density lipoprotein (LDL), and high-density lipoprotein (HDL) were performed. All the values were based on Pars Azmoon kits (Tehran, Iran). Other relevant data such as the patients’ blood group were collected from their medical history and registered medical files.

The Farsi version of the Rose Angina Questionnaire was applied in our study. This questionnaire evaluates angina (angina at all, angina on exertion, and angina at ordinary pace), as well as pain response, pain relief, pain relief duration, and pain location. The intra-class correlation coefficients ranged between 0.76 and 0.98 (Cronbach’s alpha=0.65).^8^ The patients were classified by the guidelines of the London School of Hygiene and Tropical Medicine.^9^

The Minnesota coding for ECG was first presented by the University of Minnesota in 1960.^10^ This classification is very useful in cardiovascular epidemiology, although it is not applied in daily practice. In our study, we included ECG records with a diagnostic Q-code (Minn. code 1-1-1 through 1-2-5 plus 1-2-7) and ECG records with ST-segment elevation code 9-2 PLUS (T-wave inversion code 5-1 or 5-2 in the absence of 7-2-1 or 7-4) as our definite diagnosis. 

Furthermore, ECG records with an equivocal Q-code (any 1-3-code), ECG records with ST-segment depressions (code 4-1-x or 4-2 or 4-3 in the absence of 72-1 or 7-4), ECG records with T-wave inversions (code 5-1 or 5-2 or 5-3 in the absence of 7-2-1), and ECG records with ST-segment elevations (code 9-2) were considered as probable CAD.

All the collected data were analyzed by using SPSS, version 12. The data are presented as means±standard deviations (SDs), ranges, and percentages. The mean of studies variables was compared by t-test and the qualitative variables was compared by Chi- square. Pearson correlation was applied to assess the relationship between variables. A p value less than 0.05 was considered statistically significant.

## Results

The study sample consisted of 412 men and 389 women at a mean age of 54.82±12.11 years. Furthermore, 592 individuals were from urban districts, while the others were from the rural areas. The anthropometric data are presented in Table 1.

**Table 1:** Demographic information of the study population

Parameters	Female	Male	P
Age (y)	50.81±13.19	58.22±11.11	<0.001
Weight (kg)	67.34±13.38	72.14±14.12	<0.001
Height (cm)	154.8±6.28	168.26±7.99	<0.001
Waist circumference (cm)	94.64±11.78	90.25±11.03	<0.001
Hip circumference (cm)	101.17±9.95	96.70±8.90	<0.001
Body mass index (kg/m^2^)	28.06±4.85	25.42±4.58	<0.001

In the physical examination, the mean values of the systolic and diastolic blood pressures were 127.09±21.53 and 79.49±11.79 mmHg, respectively. In total, 8 subjects had increased jugular venous pressures, 2 suffered from central cyanosis, and 10 presented with clubbing. Fifty-four individuals had abnormal pulmonary auscultation (21 fine crackles, 16 cases of wheezing, and 29 coarse crackles), and 74 subjects had abnormal pathologic heart sounds.

**Figure 1:**
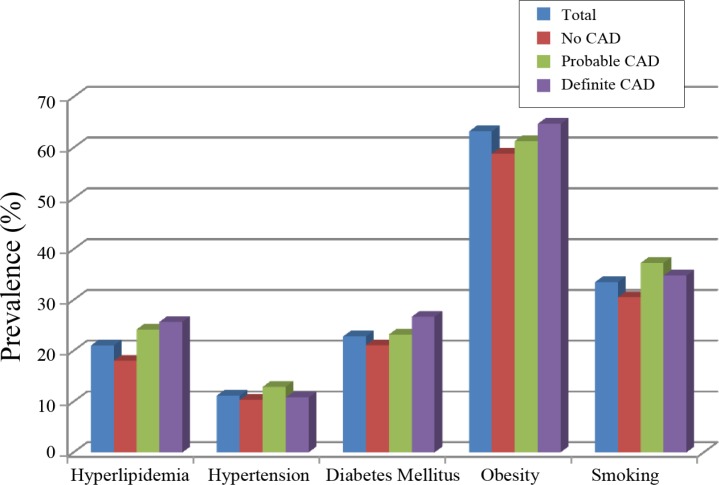
Prevalence of major cardiac risk factors in the included patients.

Based on the patients’ past medical history, 22.2% of the subjects had a prior history of smoking, 64% had the experience of at least 1 episode of dyslipidemia, 17.4% had a documented history of type 2 diabetes, and 23.3% had normal blood pressures. The major risk factors are presented in Figure 1.

CAD, Coronary artery disease

Based on the Rose Angina Questionnaire, 19% of the subjects had either typical (7.5%) or atypical angina (12.5%) (Table 2). Regarding the Minnesota ECG codes, 14.4% and 9.1% of the participants had probable and definite CAD, respectively. According to the individuals' medical files, 9.4% of the patients used cardiovascular medications for CAD, 9.9% had a prior history of admission to the CCU or the cardiac ward, 3% had a definite diagnosis of myocardial infarction, 2.4% had abnormal exercise-stress-test results, and 3.2% had abnormal angiographic results. By eliminating the common cases, 15.7% of the patients were diagnosed as definite and 18.7% as probable CAD.

**Table 2:** Final diagnosis of coronary artery disease by the Rose Angina Questionnaire, Minnesota ECG codes, and documented history (separately and in total)

	Rose Angina Questionnaire	Minnesota Codes	Document- ed History	Total
Healthy	645 (80.5)	613 (76.5)	-	625 (78.0)
Probable CAD	96 (12.1)	115 (14.4)	-	150 (18.7)
Definite CAD	60 (7.5)	73 (9.1)	72 (9.1)	125 (15.7)

Data are presented as n (%)

ECG, Electrocardiogram; CAD, Coronary artery disease

No significant difference was found in the distribution of probable (29.8% of the men vs. 34.2% of the women) and definite CAD (17.1% of the men vs. 19.9% of the women) between the male and female patients (p value=0124). Similarly, no significant difference was reported in the prevalence of probable and definite CAD between the urban and rural residents. In total, 31% and 35% of the study population had probable CAD in the rural and urban regions, respectively. Additionally, 18.2% and 20.1% of the subjects had definite CAD in the rural and urban regions, respectively (P=0.347). Table 3 presents the demographic data and biochemical assays of all 3 groups. As is indicated, triglyceride (P=0.001), total cholesterol (P=0.029), HDL (P=0.024), LDL (P=0.012), and blood urea nitrogen (P=0.025) were significantly different among the three groups. In addition, there was a significant difference between the probable CAD and no CAD groups in terms of blood urea nitrogen (P<0.05).

**Table 3:** Laboratory test results in all 3 group of the subjects*

	No CAD	Probable CAD	Definite CAD	Total	P
TG (mg/dL)	147.52±107.64	154.51±105.87	199.10±133.74	161.17±112.17	0.001
Total Chol. (mg/dL)	190.00±40.36	193.01±37.70	200.91±44.83	192.23±40.71	0.029
HDL (mg/dL)	46.26±7.11	44.82±6.53	42.55±7.09	44.11±7.06	0.024
LDL (mg/dL)	105.15±38.17	117.95±37.06	131.98±43.29	116.71±38.81	0.012
CRP (mg/dL)	5.96±7.70	6.41±7.60	6.59±9.26	6.14±7.95	0.665
BUN (mg/dL)	15.23±5.20	16.66±8.17	15.82±4.51	15.59±5.82	0.025
Creatinine (mg/dL)	0.91±0.22	0.94±0.22	0.93±0.19	0.92±0.22	0.348
FBS (mg/dL)	110.03±37.92	114.82±50.64	114.60±44.83	111.65±41.74	0.322
Uric Acid (mg/dL)	9.93±4.10	6.75±2.72	7.10±2.96	6.93±3.71	0.704

CAD, Coronary artery disease; TG, Triglyceride; Chol, Cholesterol; HDL, High-density lipoprotein; LDL, Low-density lipoprotein; CRP, C-reactive protein; BUN, Blood urea nitrogen; FBS, Fasting blood sugar

* Data are presented as mean±SD

## Discussion

Our study indicated that CAD distribution in the Iranian city of Borujerd is in accordance with the patterns detected in other world regions (18.7% and 15.7% for probable and definite CAD, respectively), which is considerably higher than our speculations. This indicates that lifestyle changes and urbanization have exerted negative impacts on both urban and rural residents, considering their inactivity and high-calorie diets. 

A population-based study in Asia (except for India and China) during 1996 revealed that 10.1% of the population were suffering from CVDs (mortality rate=24.4%).^7^ As the Middle Eastern countries have younger populations, lower CAD rates are expected; however, CAD mortality is following an increasing trend, accounting for nearly 25% to 45% of all deaths.^6^

American health-care providers estimate that 71 million adults have at least 1 CVD, which accounts for 12 million cases of CAD and 5 million cases of heart failure. 5 Furthermore, as a previous study indicated, CAD was the most prevalent diagnosis in emergency departments.^11^ Direct and indirect costs of CAD approximate to $3000 per patient each year in the United States of America.^12^ Nonetheless, not many population-based surveys have been conducted in the Middle East countries regarding this issue. Our study indicated that CAD is prevalent in the Iranian population, compared to other regions, which can be a red flag for Iranian health-care policymakers.

Our study demonstrated CAD risk factors among the Borujerd residents. The Framingham study has provided sufficient information on this issue.^13^ Moreover, all representative studies such as the PROCAM^14^ and the Seven Countries study^15^ have shown similar results. Nevertheless, the most significant difference between our results and other similar studies is the huge decline in the age range of subjects with the same risk factors. As was previously reported, nearly 50% of middle-aged residents (35%–65%) were suspected of or diagnosed with CAD. Therefore, our young population is at a higher risk for CAD.

Our study demonstrated that 64% of the patients either suffered from dyslipidemia or had a prior history of this condition (not necessarily hyperlipidemia, which accounted for 21% of the cases). Overall, 27.1% of our study population had LDL levels between 130 and 200 mg/dL and 38.2% had LDL levels higher than 200 mg/dL. High total cholesterol, high triglyceride, and low HDL levels have also been reported in similar studies. In our study, dyslipidemia was the most frequent risk factor among the subjects. Still, other risk factors including hypertension, smoking, obesity, and diabetes mellitus were similarly reported in other studies.^13^^-^^16^

A similar study was conducted on cardiac risk factors in the Iranian provinces of Isfahan and Markazi. The obtained results were similar to the current findings. In total, 6300 individuals were included from both provinces. Overall, 34.3% and 32.2% of the subjects in the Isfahan and Markazi provinces had 1 risk factor and 19.3% and 15% had 2 risk factors, respectively. Similarly, a high LDL level was the most prevalent risk factor in both groups.^16^

Another study on 400 random samples in the Iranian city of Kerman demonstrated that 25.8% of the subjects had hyperlipidemia, 14% had triglyceridemia, 7.8% had high systolic blood pressures, and 24% presented with high diastolic blood pressures.^17^ Another study in Bushehr city on 2092 random adult subjects revealed that 97% had at least 1 risk factor for CAD; furthermore, 44.3% of the males and 69% of the females had at least 2 risk factors.^18^ A similar study in Tehran demonstrated that 21.8% of the subjects (18.8% of the men and 22.3% of the women) suffered from CAD.^19^

The most important finding of all such Iranian studies is that the prevalence of CAD in adults is higher in women than in men, although we did not find a significant difference. Be that as it may, our findings, which are inconsistent with previous studies, pose a new issue apropos CAD epidemiology in Iran. The observed discrepancy could be due to higher life expectancy and higher rates of obesity in women (reported in our study due to the subjects’ inactivity). 

Our study was an observational epidemiologic study on 795 individuals, selected via cluster random sampling in a city in the west of Iran. Comparisons between the current results and other studies conducted in other parts of the country indicate that the prevalence of CAD and its associated risk factors is similar to the rates reported in Tehran, Persian Gulf islands, Central Iran, and Western societies (Europe and the USA).

Obesity, smoking, poor lifestyle, hypertension, insulin resistance, and diabetes, which are not expected to be commonly observed in a developing country, are the major risk factors for CAD. It is required to enhance the level of and change dietary habits and lifestyle by means of mass media. The elimination of the associated risk factors is more cost-effective than the treatment of the disease.

## Conclusion

The current study demonstrated that the distribution of CAD in the Iranian city of Borujerd is significantly differ between the age groups and genders and it was revealed that obesity and smoking, hyperlipidemia, diabetes and hypertension are the most common risk factors, respectively. so, lifestyle modifications and urbanization have exerted similar negative impacts on both urban and rural residents in the studied population, mainly due to inactivity and high-calorie diets and training programs are, therefore, essential.

## Notes:

***This paper should be cited as: ***Maleki A, Ghanavati R, Montazeri M, Forughi S, Nabatchi B. Prevalence of Coronary Artery Disease and the Associated Risk Factors in the Adult Population of Borujerd City, Iran. J Teh Univ Heart Ctr 2019;14(1)1-5.
